# An integrated target field framework for point-of-care halbach array low-field MRI system design

**DOI:** 10.1007/s10334-023-01093-z

**Published:** 2023-05-20

**Authors:** Bart de Vos, Rob F. Remis, Andrew G. Webb

**Affiliations:** 1 C.J. Gorter MRI Center, Leiden University Medical Center, Leiden, Netherlands; 2 Signal Processing Systems, Delft University of Technology, Delft, Netherlands

**Keywords:** Low-Field MRI, System Design, Inverse source problem, Halbach array, Gradient coil, RF coil

## Abstract

**Objective:**

Low-cost low-field point-of-care MRI systems are used in many different applications. System design has correspondingly different requirements in terms of imaging field-of-view, spatial resolution and magnetic field strength. In this work an iterative framework has been created to design a cylindrical Halbach-based magnet along with integrated gradient and RF coils that most efficiently fulfil a set of user-specified imaging requirements.

**Methods:**

For efficient integration, target field methods are used for each of the main hardware components. These have not been used previously in magnet design, and a new mathematical model was derived accordingly. These methods result in a framework which can design an entire low-field MRI system within minutes using standard computing hardware.

**Results:**

Two distinct point-of-care systems are designed using the described framework, one for neuroimaging and the other for extremity imaging. Input parameters are taken from literature and the resulting systems are discussed in detail.

**Discussion:**

The framework allows the designer to optimize the different hardware components with respect to the desired imaging parameters taking into account the interdependencies between these components and thus give insight into the influence of the design choices.

## Introduction

Access to MRI is severely limited in many countries [1, 2]. According to the International Atomic Energy Agency the number of MRI’s per million inhabitants in Sub-Saharan Africa is 0.3, while this figure is 22.2 averaged throughout Europe and Northern America [3]. Developing low-field strength (< < 1 Tesla) systems has the potential of improving this situation, decreasing the overall cost by eliminating the need for an expensive superconducting magnet, high-power water-cooled gradient coils, proprietary and hard-to-fix electronics, and elaborate site preparation. Accessibility can also be improved by designing these low-field systems such that they can be brought to the patient (point-of-care systems). Several recent reviews on the topic of low-field MRI have been published [4–8].

Multiple groups have successfully performed *in-vivo* experiments with point-of-care systems. In the summary below, individual image datasets were typically acquired in ~ 10 min. Popular point-of-care magnet designs are the H- and C-type parallel plate systems, where the letter refers to the shape of a yoke made of a material with high permeability, connecting the plates containing the permanent magnets. Nakagomi et al*.* [9] designed and constructed an H-type magnet weighing 200 kg, with an average field strength of 200 mT measured in a 100 mm diameter spherical volume (DSV). The system was mounted and operated from inside a mini-van. Elbow images were obtained using a field-of-view (FOV) of 180 × 180 mm^2^ and a digital resolution of ~ 0.7 × 1 mm^2^, using 9 slices with a thickness of 3 mm. He et al*.* [10] created a similar H-type system for a larger imaging volume (200 mm DSV), and showed *in-vivo* images of stroke patients using a 350 kg, 50.9 mT magnet. The images obtained with this system had a slice thickness of 10 mm and the FOV used was 260 × 260 mm^2^ with a resolution of ~ 1.5 × 2 mm^2^. The company Hyperfine has built an FDA approved 64 mT, C-type system, with the magnet weighing 320 kg. The reported obtainable resolution is ~ 1.5 × 1.5 × 5 mm^3^. The system has been used at the bedside for adult neuroimaging [11–13] and paediatric neuroimaging [14], it has been operated from within a mini-van [15], and has been used in a resource-constrained environment [16]. Using an open-access dataset the diagnostic performance of this device has been simulated and compared to a clinical 3 T system [17]. A similar system for adult neuroimaging was designed recently by Liu et al*.* [18], which acquired all images with a digital resolution of approximately 2 × 2 × 10 mm^3^. The system without amplifiers weighed 750 kg, and had a field strength of 55 mT measured over a 240 mm DSV. Both the Hyperfine system and the system designed by Liu et al*.* implemented multiple external antennas which were used to reduce the effects of any electromagnetic interference. For educational purposes, rather than imaging a body region Cooley et al*.* [19] designed a table top parallel plate system with a 1 cm FOV. Reproducibility and value beyond medical applications were demonstrated by building 20 of these systems.

Other groups have proposed more lightweight solutions using a magnet based on a discrete Halbach array. These systems consist of rings holding thousands of small magnets positioned in a configuration first described by Halbach [20–22], and creating a B_0_ field transverse to the bore. Blümler [23–26] created multiple magnet systems using an array of identical bar magnets oriented such that it resembles the magnetisation described by Halbach. Cooley et al. [27, 28] designed an MRI system weighing 280 kg with a gradient in the main magnetic field, and an average field strength of 80 mT measured over a 200 mm DSV. Brain images with a resolution of 2.2 × 1.3 × 6.8 mm^3^ were acquired with a FOV of 200 × 200 × 200 mm^3^. Electromagnetic interference was reduced using multiple noise antenna’s and an algorithm that uses the impulse response of the sensor data to remove the interference in k-space [29]. In a more recent study the visibility of lesions in the brain were compared to a 1.5 T clinical system [30]. O’Reilly et al*.* [31, 32] designed a magnet weighing approximately 75 kg with a field strength of 50 mT measured over a 200 mm DSV. Conventional Fourier encoding methods using three gradients were employed. Images of the knee with 2 mm isotropic resolution and brain images with a resolution of 4 × 4 × 4 mm^3^ were shown. A similar 70 mT, 200 kg magnet system designed for extremity imaging was created by Guallart-Naval et al*.* [33]. Wrist and hand images were acquired using a 200 × 160 × 80 mm^3^ FOV with a digital resolution of 0.8 × 1.3 × 5 mm^3^.

There are relative advantages and disadvantages associated with each of the approaches to magnet design. A parallel plate design is less involved since it consists simply of large pieces of permanent magnet material and a yoke. In addition, it is more open than the Halbach array which is a closed cylinder. However, the Halbach system produces the maximum field strength per unit weight of magnetic material and construction is considered to be safer due to the magnetic forces being divided over thousands of small magnets. Gradient coils for the parallel plates must be planar in nature, which are intrinsically less efficient than the cylindrical coils used in a Halbach array. Radiofrequency (RF) coil design is similar, although coupling to the gradient coils is higher in the Halbach design due to the cylindrical geometry.

The overall system dimensions are crucial to facilitate point-of-care since designing the magnet, gradient and RF coils to be as compact as possible is essential for portability. The minimum physical dimensions are ultimately governed by the targeted application. However, there are well known trade-offs between size and performance. Magnets with a smaller diameter bore produce higher magnetic field strengths and are lighter, but have a poorer B_0_-homogeneity over a given field-of-view [31]. Gradient coils with smaller diameter have higher efficiency, and a smaller length/diameter ratio results in poorer linearity [34]. Higher image spatial resolution requires higher gradient strengths with respect to the B_0_-inhomogeneity. Similarly, RF coils with smaller length/diameter have higher transmit/receive efficiency, but poorer RF homogeneity [35].

In this work we introduce an iterative design approach which integrates the different hardware designs (magnet/gradient coils/RF coil) into one framework. Using time efficient methods allows the designer to obtain a fast understanding of the interdependencies of the input parameters to change to obtain desired imaging specifications. As a consequence the user can design optimal point-of-care systems, where optimal corresponds to the most compact solution, which is able to obtain the desired image characteristics with the maximum B_0_ field strength for the highest SNR. Doing this via a linear design approach, where the components are designed individually, is sub-optimal due to the many interdependencies. Therefore, we propose a framework which iterates target field methods for gradient, RF and magnet design, where desired fields are prescribed in a region of interest and the inverse source problem is solved in a semi-analytical manner. The advantage of target-field methods is that they have short computation times and can be programmed on a single platform: such methods have not been shown for magnet design, but are often used in gradient, RF- and shim coil design [36–41]. The framework incorporates the interdependencies of the hardware components and their effects on the imaging parameters and uses the gradient and RF amplifier characteristics to create a system which is as compact as possible while corresponding to pre-set imaging requirements. Halbach array based magnets are considered, where ring-diameter and ring-spacing are optimised for homogeneity and where the individual magnet size, remanence and spacing between them are fixed quantities defined by the user.

## Methods

First the framework is discussed followed by a detailed description of the individual design methods of which it consists.

### Target field framework for iterative system design

**Fig. 1:**
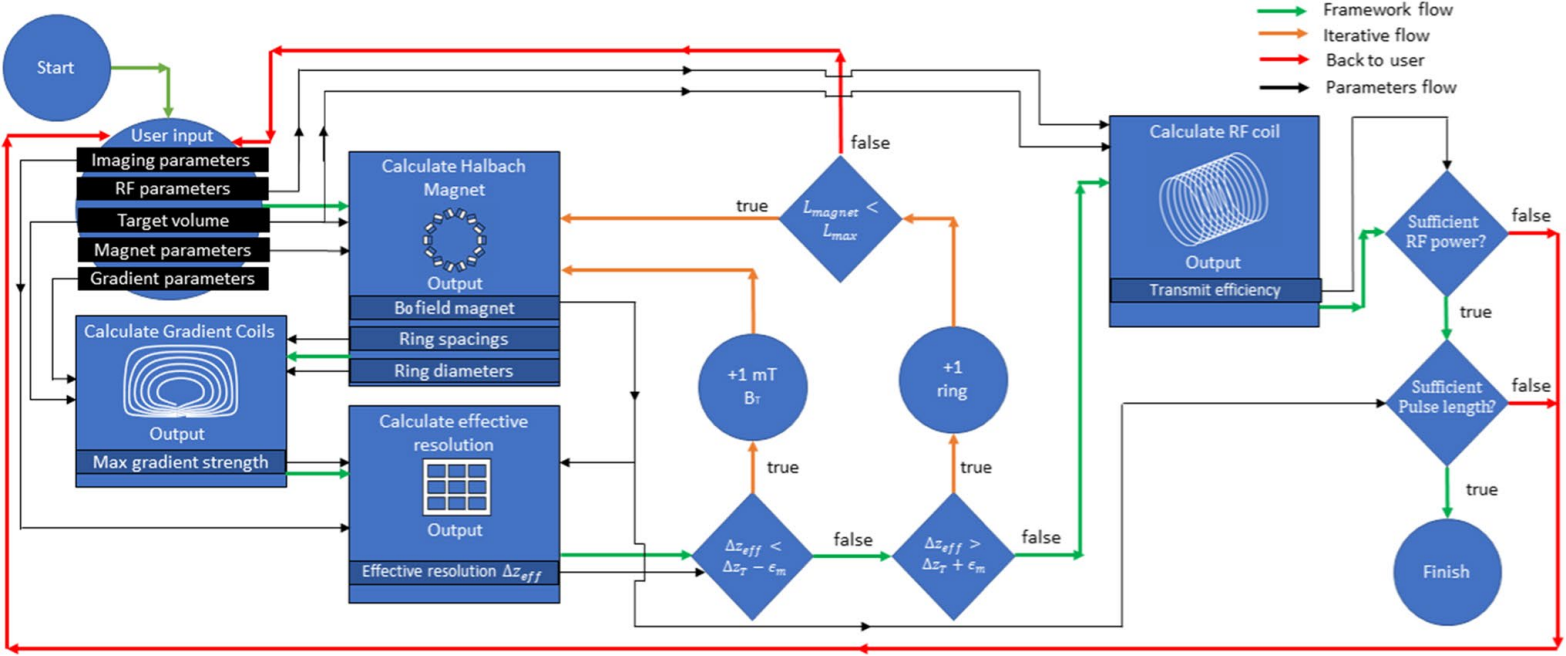
Flow chart of the system design framework. The black rectangles represent the input parameters. The light blue blocks hold the different computational methods, the dark blue rectangles positioned inside the methods show the output parameters, the diamonds represent the if-statements and the circles express the actions exerted by the framework. The green arrows indicate the natural flow of the framework, the orange arrows indicate feedback. Red arrows indicate when interaction with the user is required

A flowchart of the complete design framework is shown in Fig. 1. The computational blocks are connected with arrows where the green arrows denote the natural flow of the framework. The orange arrows show the iterative paths which are taken to revisit certain design blocks to improve the overall design. The red arrows shows when a certain limit defined by the user is reached. This will terminate the design loop. Finally the black arrows show the flow of parameters from one design block to the next. These parameters are specified in Table 1.

**Table 1:** Input parameters framework

Quantity	Head system	Extremity system
**Imaging parameters**		
Target resolution, $\Delta {z}_{T}$ [mm]	3 × 3 × 3	1.75 × 1.75 × 1.75
Target resolution margin, ${\epsilon }_{m}$ [mm]	0.1	0.01
Digital resolution [mm]	2.5 × 3 × 3	1.60 × 1.75 × 1.75
Field-of-view [mm^3^]	200 × 200 × 200	200 × 120 × 120
Data matrix	80 × 66 × 66	125 × 68 × 68
**Magnet parameters**		
Target Volume [mm^3^]	200 (DSV)	60 × 200 (r × L Cylinder)
Minimum magnet radius, ${\mathbf{r}}_{\mathrm{min}}$ [mm]	150	100
Maximum magnet length, ${\mathrm{L}}_{\mathrm{max}}$ [mm]	550	500
Minimum field strength [mT]	45	80
Number of radial magnet layers, *L*	2	2
Magnet size [mm^3^]	12	12
Azimuthal magnet spacing, $\kappa$ [mm]	20	20
Radial magnet spacing, $\delta$ [mm]	20	20
Magnet remanence, ${M}_{0}$ [T]	1.3	1.3
**Gradient parameters**		
Target Volume [mm^3^]	200 (DSV)	60 × 200 (r × L Cylinder)
Max grad amplifier current [A]	30	30
Gradient wire diameter [mm]	1.5	1.5
Min wire spacing [mm]	1	1
Max linearity error,$\sigma$	5%	5%
RF parameters		
Target Volume [mm^3^]	200 (DSV)	60 × 200 (r × L Cylinder)
RF coil length [mm]	240	240
RF coil diameter [mm]	240	140
Max RF uniformity error	20%	20%
Min RF pulse length [µs]	50	50
RF amplifier gain [dB]	54	54
Max spectrometer output [dBm]	0	0

Starting at the top left of the framework, the user specifies the input parameters for RF, magnet and gradient design, together with the desired FOV and spatial resolution. Given these, a system is designed which is as compact as possible, has the strongest possible magnetic field and has an effective resolution corresponding to the imaging requirements.

**Fig. 2:**
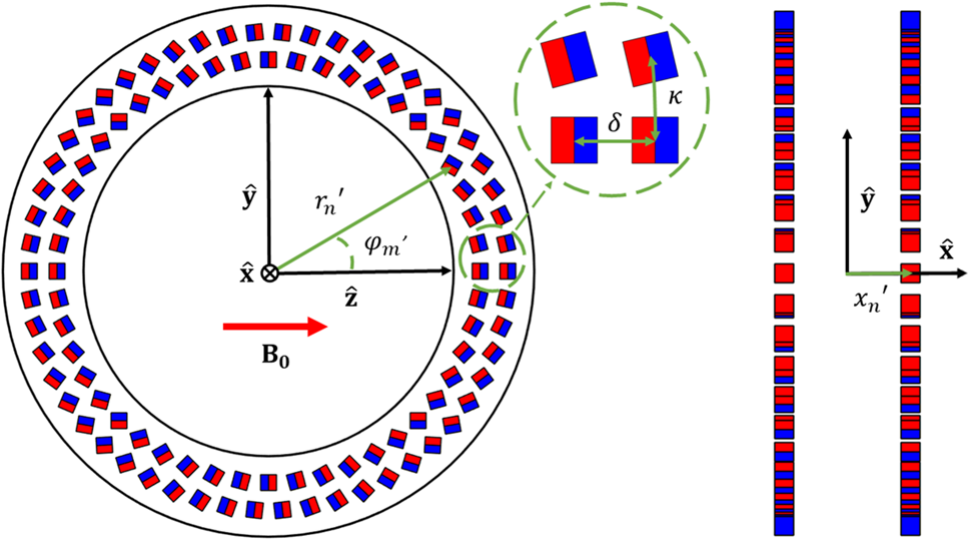
Illustration of a Halbach array depicting the coordinate system and symbols used. Left: front view of a single ring with two magnet layers. Right: side view of two rings

The first computation is that of the initial magnet design. The magnet systems considered are variable ring-diameter and ring-spacing Halbach arrays, where the individual magnets are placed in discrete rings. Such a setup is shown in Fig. 2. The initial number of rings is set by the user and is fixed within the magnet optimisation algorithm. The other starting conditions are the minimum bore diameter, minimum target field strength, volume of interest and the remanence and spacings of the individual magnets: these are all fixed throughout the framework. Based on these parameters the diameters and spacings of the rings are optimised to approach the user-specified target field while maximising homogeneity.

Next, power optimised gradient coils are designed. Their lengths and radii are determined by the magnet dimensions from the previous magnet optimisation. The most efficient coils are determined given a user-specified linearity.

Subsequently, the gradient-limited effective spatial resolution in the readout direction $\Delta {z}_{eff}$ is determined using the efficiency of the least efficient gradient coil, the maximum gradient amplifier current, the ${\mathrm{B}}_{0}$ field inhomogeneity of the magnet, and the field-of-view and number of complex data points defined by the user.

If the effective resolution is larger than the predefined threshold the homogeneity of the magnet needs to be increased. In this case the model loops back to the magnet design, and an additional ring is added. The ring-diameters and separations are again optimised creating a more homogeneous (but larger) magnet. The gradient coils are then redesigned given the new magnet geometry. Adding rings increases the allowable length of the gradient coils which increases the linearity. However, the efficiency given a certain linearity has an optimum. If increasing the length of the coils during the framework iterations decreases the efficiency, the previous shorter solution is taken.

The process of magnet and gradient design continues to iterate until the requirements for spatial resolution are met. At this point, the framework determines if it is possible to increase the target magnetic field strength ${\mathrm{B}}_{\mathrm{T}}$, while maintaining the required spatial resolution. Increasing ${\mathrm{B}}_{\mathrm{T}}$ effectively decreases the average ring-diameter and spacing, resulting in a smaller but less homogenous magnet.

This iterative process of changing the magnet design by adding rings or increasing the field strength, computing the resulting gradient coils and determining the effective resolution, is repeated until the desired effective spatial resolution is reached with the strongest possible field strength.

In the final step a solenoidal RF coil with variable wire spacing is designed: a solenoid is chosen due to the intrinsically higher sensitivity compared to saddle coils. The coil dimensions are defined by the user and optimised for transmit efficiency given a user-specified non-uniformity over the FOV. The checks performed for the RF coil are twofold: the user defines a minimum pulse length (microseconds) and the framework ensures that this excites all of the hydrogen nuclei (with a range of resonance frequencies dictated by the B_0_ field inhomogeneity) within the imaging FOV. In addition, the power available from the RF amplifier combined with the transmit efficiency of the coil, given the user-specified minimum pulse duration, must be sufficient to obtain the desired flip angles. If these conditions are met the framework is finished.

The framework terminates prematurely if: (i) the pre-specified maximum length of the system (${L}_{magnet}>{L}_{max}$) is exceeded without achieving the requested effective resolution, (ii) if the required RF power is not sufficient to obtain the desired flip angle, or (iii) if the RF pulse is not able to excite the entire FOV. The user can utilize this information to alter the input parameters such that the design is feasible.

### Target field approach for halbach array based magnets with variable ring-diameters and -spacing

In this section, we derive an expression to describe the field created by magnets in an Halbach array with variable ring-diameter and ring-spacing. Determining the diameters and spacing such that a given prescribed target field is realized is a non-linear inverse source problem which is solved using a Newton optimisation scheme.

The individual magnets of the Halbach array are modelled as magnetic dipoles. Many authors have shown that such an approximation provides a sufficiently accurate magnetic flux density approximation within the domain enclosed by the magnet array [27, 31, 42]. The magnetic flux density **B** at position $\mathbf{x}$ due to a dipole positioned at location **x**' is given by

1 $$\textbf{B}\left( \textbf{x} \right) = \frac{{\mu_{0} }}{4\pi }\left[ {\frac{{3\left( {\textbf{x} - \textbf{x}^{\prime}} \right)\left[ {\textbf{M} \cdot \left( {\textbf{x} - \textbf{x}^{\prime}} \right)} \right]}}{{\left| {\textbf{x} - \textbf{x}^{\prime}} \right|^{5} }} - \frac{\textbf{M}}{{\left| {\textbf{x} - \textbf{x}^{\prime}} \right|^{3} }}} \right],$$

where **M** $=\mathbf{M}(\mathbf{x}\mathbf{^{\prime}})$ is the magnetization vector. In Fig. 2, the magnet configuration is shown including the coordinate system and the various symbols that are used for the design of the magnet: a right handed system is used with *z* directed parallel to B_0_ and *x* parallel to the axis of the bore.

As the optimisation is defined with respect to the ring- diameters and -spacing, cylindrical coordinates ${\mathbf{x}}^{\prime} = \left\{ {r^{\prime},\varphi ^{\prime},x^{\prime}} \right\}$ are used to indicate the position of the magnets. Furthermore, the magnets are oriented such that a set of magnets within a ring form a discrete approximation of the continuous Halbach magnetization

2 $$\mathbf{M}={M}_{0}\left[\mathrm{cos}\left(2{\varphi }^{{{\prime}}}\right)\widehat{\mathbf{z}}+\mathrm{sin}\left(2{\varphi }^{{{\prime}}}\right)\widehat{\mathbf{y}}\right],$$

where ${M}_{0}$ is the remanent magnetisation of the individual magnets. This magnetization profile creates a homogenous field in the $z$-direction in the case of an infinitely long cylinder [20–22]. The magnetization vector of Eq. (2) is now substituted into Eq. (1), and only the relevant *z*-component of the magnetic flux density $\mathbf{B}$ is considered. Assuming that interactions between the magnets (i.e. any demagnetization effects) can be neglected, the field generated by the array can be found by summing the fields of each individual magnet.

The magnets are located in *N* discrete rings and each ring may consist of *L* concentric layers of magnets to accommodate for higher field strengths. The radius of the $l$ th layer in the *n*th ring is denoted by ${r}_{ln}={r}_{n}+\updelta \left(l-1\right)$, where $\updelta$ is the radial separation between the layers. The azimuthal distance between the magnets inside a layer $\kappa$ is held constant, meaning that the number of magnets per layer can be found as${M}_{ln}=\frac{2\uppi {r}_{ln}}{\kappa }$, and therefore varies as a function of the ring-radius. The azimuthal angle of the $m$ th magnet is ${\mathrm{\varphi }}_{m}=m\frac{2\uppi }{{M}_{ln}}$ and we find that.

3 $${B}_{z}\left(\mathbf{x}\right)=\frac{{\mu }_{0}}{4\pi }\sum_{n=1}^{N}\sum_{l=1}^{L}\sum_{m=1}^{{M}_{ln}}\left\{\frac{3\left(z-{z}_{lnm}^{^{\prime}}\right)\left[{M}_{\mathrm{z}}\left(z-{z}_{lnm}^{^{\prime}}\right)+{M}_{\mathrm{y}}\left(y-{y}_{lnm}^{^{\prime}}\right)\right]}{{\left|\mathbf{x}-{\mathbf{x}}^{\mathbf{^{\prime}}}\right|}^{5}}-\frac{{M}_{\mathrm{z}}}{{\left|\mathbf{x}-{\mathbf{x}}^{\mathbf{^{\prime}}}\right|}^{3}}\right\},$$

where ${z}_{lnm}^{^{\prime}}={r}_{ln}\mathrm{cos}{{\varphi }^{\mathbf{^{\prime}}}}_{m}$ and ${y}_{lnm}^{^{\prime}}={r}_{ln}\mathrm{sin}{{\varphi }^{\mathbf{^{\prime}}}}_{m}$. Using Eq. (3), the ring-diameters and -spacing can now be optimised using a target field approach. The procedure for the ring-radii is illustrated here. Ring-spacing optimisation is done in a similar manner and is therefore only briefly discussed. The desired target field values at the locations $[{\mathbf{x}}_{1},{\mathbf{x}}_{2},\cdots ,{\mathbf{x}}_{K}]$ are stored in the *K* × 1 vector ${\mathbf{b}}_{T}$.

The unknown ring-radii are represented by the *N* × 1 vector $\mathbf{r}$ and the *N* × 1 vector $\mathbf{d}$ contains the ring-positions. Finally, ${\mathbf{b}}_{z}\left(\mathbf{r}\right)$ is the *K* × 1 vector obtained by evaluating (3) for every target field position and parameter vector $\mathbf{r}$. The objective now is to find vector **r** such that

4 $${{\textbf{b}}}_{T}-{{\textbf{b}}}_{z}\left({\textbf{r}}\right)=0.$$

This is clearly a non-linear problem, and a Newton optimization scheme [43] is chosen to find a vector **r** for which Eq. (4) is (approximately) satisfied. Specifically, starting with an initial guess **r**_0_, the optimal vector is found in an iterative manner using the update equation

5 $${{\textbf{r}}}_{i+1}={{\textbf{r}}}_{i}+{\mathcal{J}}_{r}^{+}\left[{{\textbf{b}}}_{T}-{{\textbf{b}}}_{z}\left({{\textbf{r}}}_{i}\right)\right].$$

Here ${\mathcal{J}}_{\mathrm{r}}^{+}$ is the pseudo inverse of the *K* x *N* Jacobian matrix which is given by

6 $${\mathcal{J}}_{r}=\left[\begin{array}{ccc}\frac{\partial {B}_{z}\left({{\textbf{x}}}_{1},{r}_{1}\right)}{\partial {r}_{1}}\,\,\,& \,\,\,\cdots & \frac{\partial {B}_{z}\left({{\textbf{x}}}_{1},{r}_{N}\right)}{\partial {r}_{N}}\\ \vdots \,\,\,&\,\,\, \,\,\,\ddots \,\,\,&\,\,\, \vdots \\ \frac{\partial {B}_{z}\left({{\textbf{x}}}_{K},{r}_{1}\right)}{\partial {r}_{1}}\,\,\,&\,\,\, \cdots \,\,\,&\,\,\, \frac{\partial {B}_{z}\left({{\textbf{x}}}_{K},{r}_{N}\right)}{\partial {r}_{N}}\end{array}\right],$$

To obtain practical solutions for the clear bore of the magnet, lower bounds on the ring-radii are imposed and the radii are set to the predefined minimum value if the condition is violated. This minimum value is also taken as an initial guess for all ring -radii and defines the starting vector ${\mathbf{r}}_{0}$. In the case of a homogenous target field positioned at the centre of the magnet, mirror symmetry of the ring diameters and distances can be utilized with respect to the $x=0$ plane. Finally, the iterative process is terminated if the relative error falls below a specified tolerance or a maximum number of iterations is reached.

The ring-distance is optimised in a similar fashion, only requiring Eqs. (4–6) to be redefined in terms of the ring-positions *x*′. Lower bounds on the ring distance are applied to take the thickness of the rings into account. To fix the magnets in the rings plastic lids are typically used: the minimum practical thickness is ~ 0.5 mm for each lid. Therefore a spacing of 1 mm between the rings is chosen as a starting point.

Because the field strength is more sensitive to small changes in the ring-radii than to changes in the ring-spacing [25, 31], the ring-radii optimization is performed before ring-spacing optimization. The ring-spacing optimization starts with the best result of the ring-radii optimization, effectively focusing primarily on homogeneity and not on the field strength.

The following iterative algorithm for the magnet design summarizes the discussion above:
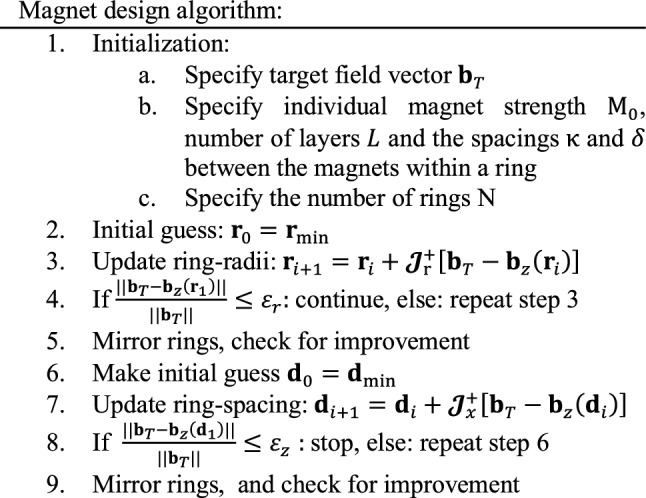


### Power optimised gradient coils

The gradient coils are designed using a previously implemented (quasi-static) target field approach [38–41]. The most important equations are summarized below, as well as the design choices which are integrated into the framework.

The target field method finds a continuous current density represented by a weighted sum of sinusoidal basis functions which create a prescribed gradient field where the same FOV is used as for the magnet design. The wire patterns are found by taking contours of the corresponding stream function [44]. In particular, the stream function presented by Forbes and Crozier [40], namely,

7 $$\psi \left( {\varphi ,x} \right) = - \mathop \sum \limits_{p = 1}^{P} \frac{2\Lambda }{{p\pi }}\gamma_{p} \cos \left( {\frac{{p\pi \left( {x + \Lambda } \right)}}{2\Lambda }} \right) + \mathop \sum \limits_{p = 1}^{P} \mathop \sum \limits_{q = 1}^{Q} \frac{2\Lambda }{{p\pi }} [ \alpha_{pq} \cos \left( {q\varphi } \right) + \beta_{pq} \sin (q\varphi )] \sin \left( {\frac{{p\pi \left( {x + \Lambda } \right)}}{2\Lambda }} \right)$$

is used for gradient design. The above equation is defined for − $\Lambda$ $<x<\Lambda$, where 2 $\Lambda$ is the length of the coil, and ${\alpha }_{pq}$, ${\beta }_{pq}$ and ${\upgamma }_{p}$ are unknown expansion coefficients. *P* and *Q* determine the number of harmonic modes that are taken into account and if this number is sufficiently large then the sum of the trigonometric basis functions weighted by the expansion coefficients gives an accurate representation of the surface current for any chosen target field. The ideal weighting is found by substituting the corresponding equations for the current density into Biot-Savart's law and rewriting the resulting expressions as a system of equations for the unknown expansion coefficients. The desired expansion coefficients are obtained by solving this typically ill-conditioned system (see Eq. (8))

Using prior knowledge about the desired coil structure and the target fields, the number of unknowns (number of expansion coefficients) can be significantly reduced. Specifically, to realize *x*- and *z*-gradient coils, only the expansion coefficients ${\alpha }_{pq}$ are required, while for *y*-gradients only the ${\beta }_{pq}$ coefficients need to be taken into account.

As mentioned above, the system of equations for the expansion coefficients is typically ill-conditioned and requires regularisation. Specifically, for each gradient coil, the expansion coefficients are determined by minimizing the regularised least squares functional

8 $$\mathcal{F}\left( {\textbf{a}} \right) = \frac{1}{2}\left| {\left| {{\text{W}\textbf{a}} - {\textbf{k}}} \right|} \right|_{2}^{2} + \frac{1}{2}\lambda {\textbf{a}}^{{\text{T}}} \Upsilon {\textbf{a}}.$$

Here, W is the system matrix representing the Biot-Savart relationships of the transverse field component, $\mathbf{a}$ is the vector containing the unknown expansion coefficients, $\mathbf{k}$ is the target field vector and $\Upsilon$ is a diagonal matrix containing coil efficiency parameters described in [41]. In case of gradient coil design, the regularisation parameter $\uplambda$ controls the trade-off between gradient efficiency ([TA^−1^ m^−1^]) and how well the desired linear target field is produced. Performing a sweep of this regularization parameter results in gradient coils with different values for these two performance metrics. The maximum linearity error is determined using.

9 $$\sigma =max\left\{\left|\frac{{\mathrm{G}}_{T}(\mathbf{x})-G(\mathbf{x})}{{G}_{T}(\mathbf{x})}\cdot 100\right|\right\}.$$

Where, ${\mathrm{G}}_{T}(\mathbf{x})$ is the targeted field and $\mathrm{G}\left(\mathbf{x}\right)$ is the realised field. In general, the efficiency and linearity error increase with higher values of $\uplambda$, choosing a maximum allowable error thus results in the most efficient coil. The number of turns influence the gradient efficiency. This number is taken such that a minimum specified wire spacing is reached. The value of the gradient target field strength can be chosen arbitrarily as the resulting value of the stream function and thus the required current going through the wires will scale with it.

The maximum length and radius of the gradient coils are determined by the dimensions of the magnet. The $y$- and $z$-gradients have the same wire patterns but are rotated by $45^\circ$ with respect to each and other. Due to the target field structure combined with the coil geometry these gradient coils are intrinsically more efficient than the $x$-gradient coils, and require a reduced length to obtain a specified linearity. Given these observations, the $x$-gradient is chosen to be the inner coil followed by the $y$- and $z$-gradients. It can occur that, depending on the number of magnet layers and the target field strength, one or both of the transverse gradient coils do not fit inside the magnet. In this case it is possible to place the gradient coil outside the magnet structure, which has been proposed in [45], but brings a significant efficiency penalty.

Finally, the gradient coils resulting from this method do not require active shielding due to the absence of a cryostat which is conventionally the main structure in which eddy currents are induced. Wire wound gradient coils are chosen, due to their relative ease of construction using 3D printed moulds. The following design algorithm is followed for the gradient coils.
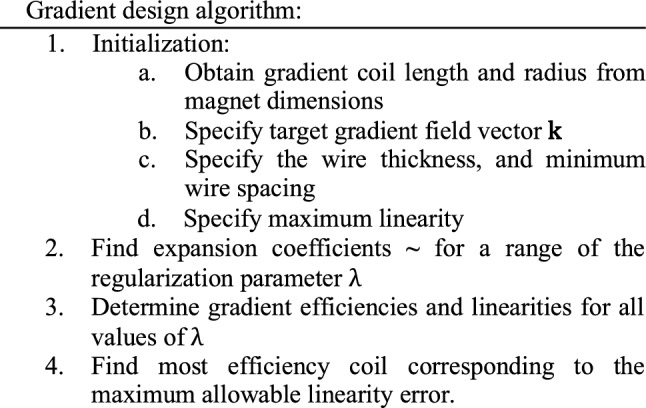


### Target field RF coils

The same approach taken for the gradient coils is used to obtain wire patterns for the RF coils. A quasi-static field approach has proven to be appropriate for the design of the field pattern [45]. A solenoid with distributed wires is obtained by taking a uniform target field throughout the volume with the field directed along the axis of the bore. Only the first term ${\upgamma }_{p}$ from Eq. (7) needs to be taken into account. Subsequently, the functional Eq. (8) can be constructed, with $\mathrm{W}$ now containing the Biot-Savart relationships corresponding to the axial field direction. The regularisation parameter controls a trade-off between the transmit efficiency and how well the field is produced within the target region. The user specifies a certain uniformity error, which results in the particular wire pattern. By specifying the target volume and the allowable RF uniformity error, the resulting field, though a quasi-static simulation, is representative for a B_1_^+^ pattern. The frequency-spread in the B_0_ field gives an indication of the coil bandwidth that can be obtained while still exciting all frequencies in the target volume. The finite support of the hard RF pulse results in a sinc function in the frequency domain. The FWHM of the central lob of the sinc function should cover the spread in frequencies created by the B_0_ field inhomogeneities. The minimum desired pulse length defined by the user is used to check if the flip angles can be achieved given the simulated transmit efficiency and the RF amplifier capabilities. Hard RF pulses used for volume excitations and refocussing are considered and the maximum desired flip angle is set at 180 degrees. The transmit efficiency is computed using 1 mm copper wire.

### Image spatial resolution

The effective spatial resolution of the image is represented by the point spread function (PSF) which is different in the frequency and phase encoding directions. Here we assume that three-dimensional images are acquired using one frequency and two phase encoding gradients. The PSF in the frequency encoding direction depends upon the maximum gradient strength, the ${\mathrm{B}}_{0}$-field inhomogeneity, and the digital resolution (FOV divided by the number of data points acquired): in the phase encoding directions the digital resolution is the only factor that needs to be considered. The resolution in the frequency encoding direction is affected by the exponential ${T}_{2}^{*}$ decay, which has a blurring effect. The linewidth is assumed to be a Lorentzian, such that $\frac{1}{{T}_{2}^{*}}= \frac{1}{{T}_{2}}+{\gamma\Delta B}_{0}$ [46], where ${T}_{2}$ is the transverse relaxation time, and the second term consists of the product of the gyromagnetic ratio $\gamma$ and $\Delta {B}_{0}$, the voxel field variation. The assumption is made that the field inhomogeneities dominate $\left({\gamma\Delta B}_{0}\gg \frac{1}{{T}_{2}}\right)$, such that ${T}_{2}^{*}\approx \frac{1}{{\gamma\Delta B}_{0}}$. This assumption is valid for typical ${T}_{2}$ times encountered during *in-vivo* experiments combined with the inhomogeneities of typical Halbach systems which are hundreds to thousands of parts per million (ppm) [47]. Taking the sum of the individual phase differences caused by the inhomogeneities throughout the region of interest results in an exponential decay which goes to zero in the limit

10 $$\underset{t\to \infty }{\mathrm{lim}}\sum_{\mathbf{x}}{e}^{-i\gamma \left[{B}_{0}+\Delta B\left(\mathbf{x}\right)\right]t}\to 0.$$

Its Fourier transform is a Lorentzian. Given the situation that the magnet field inhomogeneities are the dominant term, ${T}_{2}^{*}\approx \frac{1}{\pi FWHM}$, where FWHM is the full width half maximum of the Lorentzian. The effective resolution due to the ${T}_{2}^{*}$ filtering effect [48] can be expressed as

11 $$\Delta {z}_{eff}= {N}_{z}\mathrm{\Delta z}\frac{\mathrm{tanh}\left(\frac{ \mathrm{\pi FWHM}}{2BW}\right)}{1-{e}^{- \frac{\mathrm{\pi FWHM }{N}_{z}}{2BW}}}$$

where $BW$ is the maximum obtainable readout bandwidth of the gradient system determined with the least efficient gradient, $\mathrm{\Delta z}$ the digital resolution and ${N}_{z}$ the number of points.

### Example systems

To demonstrate the potential of the framework, two distinct systems are designed. The first is a system suitable for adult neuroimaging with input parameters guided by values from the literature [10, 12, 18, 28, 32]: FOV = 200 × 200 × 200 mm^3^, isotropic effective spatial resolution ~ 3 × 3 × 3 mm^3^, data matrix 80 × 66 × 66 corresponding to 0.5 ± 0.1 mm PSF blurring in the readout direction. The minimum field strength is set to 45 mT, slightly lower than previous systems. To ensure the head can reach the centre of the magnet, taking into account the shoulders of the subject, the maximum bore length cannot exceed 550 mm and the minimum bore diameter is set at 300 mm. This ensures sufficient space for the RF coil which has a diameter and length of 240 mm, equivalent to solenoidal coils designed for previous low-field systems [9, 10, 32].

The second system is designed for imaging of the hand and wrist. A cylindrical target volume of length 200 mm and diameter 120 mm is used. The RF coil has a length of 240 mm and a diameter of 140, the same ratio with respect to the target volume as the neuroimaging system. Based on previous literature [9, 33] we used a target isotropic resolution of 1.75 mm, obtained with a 200 × 120 × 120 mm^3^ FOV and 125 × 68 × 68 datapoints, i.e. a maximum 0.15 ± 0.01 mm blurring PSF in the readout direction is allowed. The minimum field strength is set at 80 mT. The maximum length of the system is set to 500 mm to allow positioning of the wrist at the centre of the magnet.

Both systems are designed using identical RF and gradient amplifiers, again with specifications based on previous designs [41]. The RF amplifier has a gain of 54 dB combined with a spectrometer which can output a maximum 1 mW. The gradient amplifier has a maximum current output of 30 A. The maximum gradient linearity error is set at 5% throughout the target region. The maximum allowable RF non-uniformity is set at 20% and the minimum pulse length to 50 µs. The individual cubical magnets used have a remanence of 1.3 T and are 12 mm^3^. A summary of the input parameters used for the design of both systems are shown in Table 1.

## Results

### Neuroimaging system

The initial magnet setup consists of 25 rings and 20 mm ring-spacing, resulting in a length/diameter ratio of 1.7, which is an appropriate initial estimate based on previous designs [28, 31]. In the first framework iteration a homogeneity of 4329 ppm after the ring-distance optimisation is reached. Combining this with the gradient efficiency of the least efficient *x-*gradient (0.24 mTm^−1^A^−1^), and a 30 A maximum current output, results in an effective spatial resolution of 10.3 mm. This is well above the specified 3 mm and is due to poor magnet homogeneity (4329 ppm) caused by the small length/diameter ratio. An additional ring is added to improve the homogeneity. The resulting 26 rings produce a more homogenous design (1075 ppm). The longer magnet allows the length of the weakest (*x*-) gradient to also increase, which improves its efficiency slightly from 0.24 to 0.26 mTm^−1^A^−1^. This results in an effective resolution of 4.1 mm, which is still above the pre-defined maximum. An additional ring, 27 rings in total, increases the magnet homogeneity to 381 ppm and the increase in length allows for a longer gradient coil which again increases its efficiency such that the effective resolution equals 2.6 mm, which is within the specifications. The next round of iterations determines if the static magnetic field strength can be increased to improve the SNR while still maintaining the required spatial resolution: in each iteration the field strength is increased in steps of 1 mT. Consequently, the magnet length and average diameter are decreased, making the system more compact. For this example, iteration seven of the framework gives the most compact configuration with the strongest magnetic field (49 mT) and sufficient homogeneity to obtain an effective resolution of 3 mm. Increasing the magnetic field even further would cause the effective resolution to exceed the specified maximum.

The 240 mm length and diameter solenoid has a uniformity within the FOV of 20% and a transmit efficiency of 26 µT/W. Given the 1 mW output of the spectrometer and the RF amplifier gain of 54 dB, a maximum desired flip angle of 180 degrees and a pulse duration of 50 µs, the B_1_-field required equals 234 µT and therefore 9 W is required, which is well below the maximum output power. The final check is that the 50 µs RF pulse excites a bandwidth much greater than the B_0_ inhomogeneity, which indeed is the case.

**Fig. 3:**
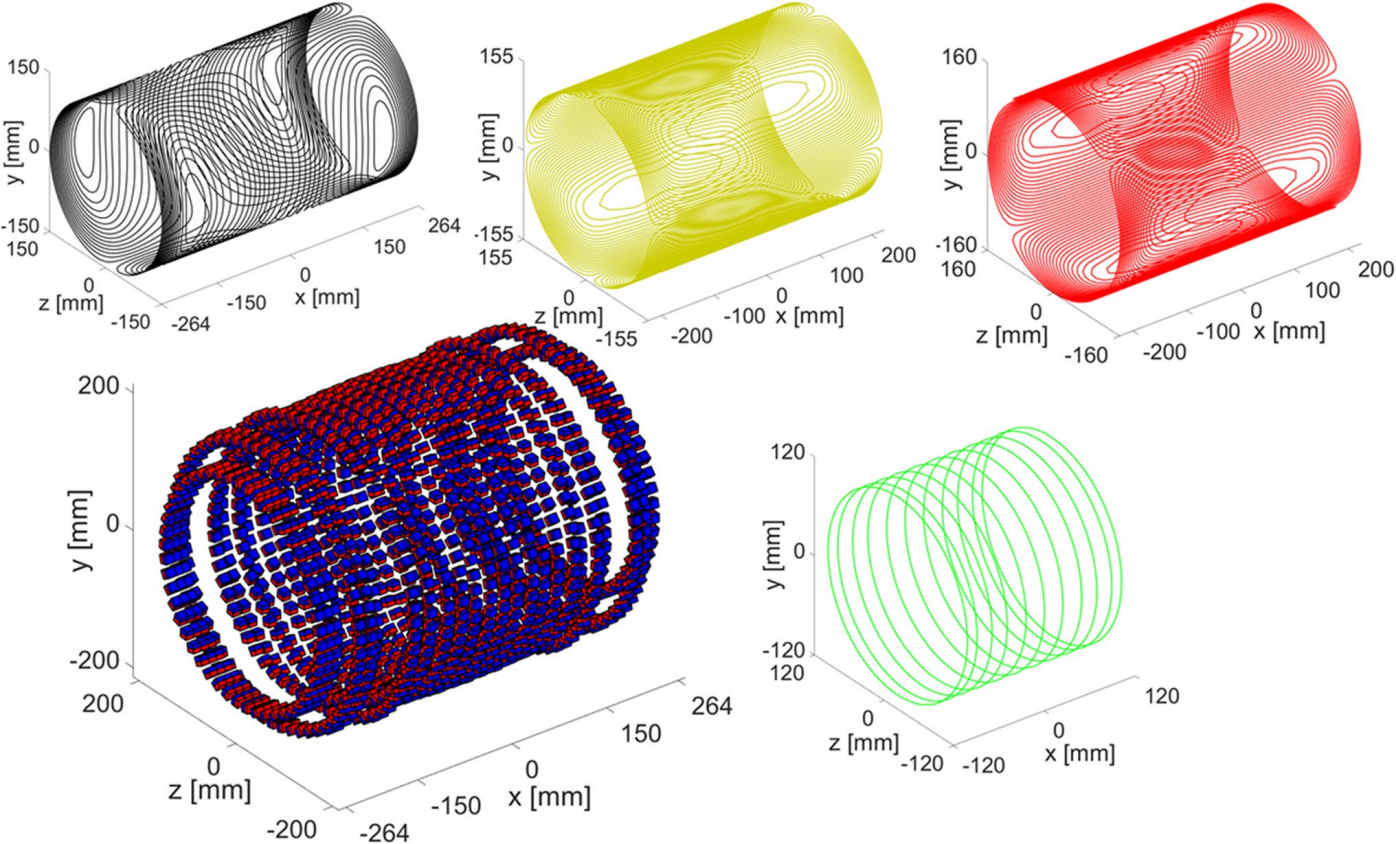
Neuro system: *x*-gradient: black, *y*-gradient: yellow, *z*-gradient: red), RF coil (green) and magnet corresponding to the final iteration of the system design framework. A single layer of magnets is shown for clarity. The values on the axes indicate the maximum dimensions

Figure 3 shows the final output of the framework, where the gradient coils, the magnet, and the RF coil are shown separately. Table 2 shows the output parameters of the framework for every iteration.

**Table 2:** Framework output parameters per iteration neuroimaging system

Framework iteration	1	2	3	4	5	6	7
**Magnet parameters**							
N rings	25	26	27	27	27	27	27
B_0_ [mT]	45	45	45	46	47	48	49
Magnet length [mm]	493	512	529	529	532	535	528
Ring radii optimisation [ppm]	6093	4773	3303	2861	3116	3210	4023
Ring spacing optimisation [ppm]	4329	1075	381	120	420	685	878
Number of magnets	3368	3532	3705	3617	3554	3485	3306
**Gradient coil parameters**							
Length x [mm]	493	512	529	529	529	529	528
Length y [mm]	437	457	475	475	475	475	450
Length z [mm]	457	457	457	457	457	457	450
Radius x [mm]	150	150	150	150	150	150	150
Radius y [mm]	155	155	155	155	155	155	155
Radius z [mm]	160	160	160	160	160	160	160
Efficiency ${\upeta }_{\mathrm{x}}$ [mTm^−1^A^−1^]	0.24	0.26	0.31	0.31	0.31	0.31	0.31
Efficiency ${\upeta }_{\mathrm{y}}$ [mTm^−1^A^−1^]	1.1	1.2	1.3	1.3	1.3	1.3	1.2
Efficiency ${\upeta }_{\mathrm{z}}$ [mTm^−1^A^−1^]	0.93	0.93	0.93	0.93	0.85	0.85	0.98
**Imaging parameters**							
FWHM [Hz]	1989	568	61	51	43	88	263
Readout resolution [mm]	10.4	4.1	2.6	2.6	2.6	2.7	3.0
**RF coil parameters**							
Transmit Efficiency [µT/W]							26
Required B_1_^+^: α = 180° [µT]							234
Maximum pulse length [µs]							545

### Extremity system

As the bore size is 1.5 times smaller than the previous example, but the volume of interest is now a cylinder which has a length relatively long compared to the diameter, the initial guess for the number of rings is chosen to be 23 with each ring holding two layers of magnets. During the first framework iteration it becomes clear that the specified field strength of 80 mT requires the centre ring-diameters to become so small that the outer gradient coil (*z-*gradient coil) does not fit inside the magnet. Due to the intrinsic efficiency difference between the *x-* and *y/z*-gradients it is feasible to place the *z*-gradient on the exterior of the magnet. The efficiency of the *z*-gradient is 1.5 mTm^−1^A^−1^, while the *x*-gradient coil has an efficiency of 0.74 mTm^−1^A^−1^ and is thus still the least efficient. The homogeneity of the magnet is 1208 ppm which results in an effective resolution of 1.88 mm, obtained with 23 rings. Since the target is 1.75 mm, the homogeneity and/or the gradient efficiency needs to be improved. This is realized by including an additional ring, which increases the homogeneity to 216 ppm and the gradient efficiency of the least efficient gradient from 0.74 to 0.82 mTm^−1^A^−1^. The effective resolution with 24 rings is 1.62 mm, which is better than required. In the successive framework iterations, the target field is increased until the value of 84 mT is reached which gives an effective resolution of 1.75 mm, computed with a homogeneity of 803 while the gradient efficiency goes from 0.82 to 0.83 mTm^−1^A^−1^.

**Fig. 4:**
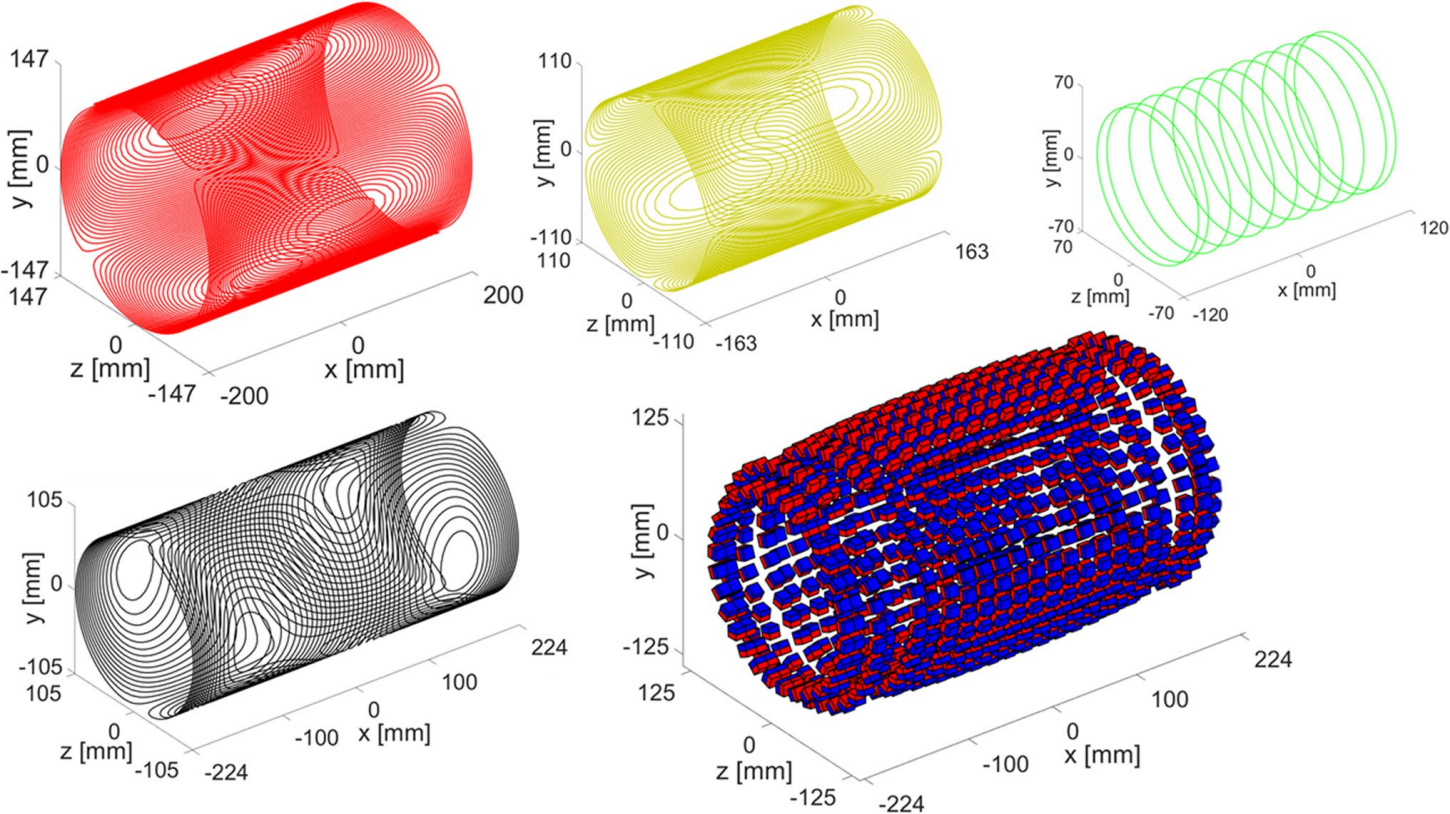
Extremity system: *x*-gradient: black, *y*-gradient: yellow, *z*-gradient: red), RF coil (green) and magnet corresponding to the final iteration of the system design framework. A single layer of magnets is shown for clarity. The z-gradient coil (red) is located at the exterior of the magnet. The values on the axes indicate the maximum dimensions

The spectrometer characteristics are the same as for the previous case. The transmit efficiency of the RF coil, designed with a diameter of 140 mm is 58 µT/W, thus 6.5 W is required for a 180° pulse. A 50 µs pulse excites the entire imaging FOV. Relevant output parameters of the framework for every iteration are shown in Table 3. Figure 4 shows the gradient magnet and RF coils at the final stage of the framework.

**Table 3:** Framework output parameters per iteration extremity syste

Framework iteration	1	2	3	4	5	6
**Magnet parameters**						
N rings	23	24	24	24	24	24
B_0_ [mT]	80	80	81	82	83	84
Magnet length [mm]	448	469	469	468	467	436
Ring-radii optimisation [ppm]	5683	2758	3035	2919	4399	3825
Ring-spacing optimisation [ppm]	1208	216	352	563	363	803
Number of magnets	1904	1990	1972	1960	1938	1922
**Gradient coil parameters**						
Length x [mm]	448	469	448	448	448	448
Length y [mm]	374	370	370	370	320	327
Length z [mm]	400	400	400	400	400	400
Radius x [mm]	105	105	105	105	105	105
Radius y [mm]	110	110	110	130	110	110
Radius z [mm]	154	154	152	151	149	147
Efficiency ${\upeta }_{\mathrm{x}}$ [mTm^−1^A^−1^]	0.74	0.82	082	0.82	0.83	0.83
Efficiency ${\upeta }_{\mathrm{y}}$ [mTm^−1^A^−1^]	2.5	2.6	2.6	2.6	2.0	2.1
Efficiency ${\upeta }_{\mathrm{z}}$ [mTm^−1^A^−1^]	1.5	1.5	1.5	1.5	1.5	1.6
**Imaging parameters**						
FWHM [Hz]	332	37	32	84	127	214
Readout resolution [mm]	1.88	1.62	1.61	1.66	1.68	1.75
**RF coil parameters**						
Transmit Efficiency [µT/W]						58
Required B_1_^+^: α = 180° [µT]						234
Maximum pulse length [µs]						348

### System comparison

The total computation time to reach the above discussed configurations is less than 15 min. A single Newton iteration takes ~ 5 s given the parameters used in the examples and using standard computing hardware (Windows, intel i3, 4-cores with 8 GB of RAM).

**Fig. 5:**
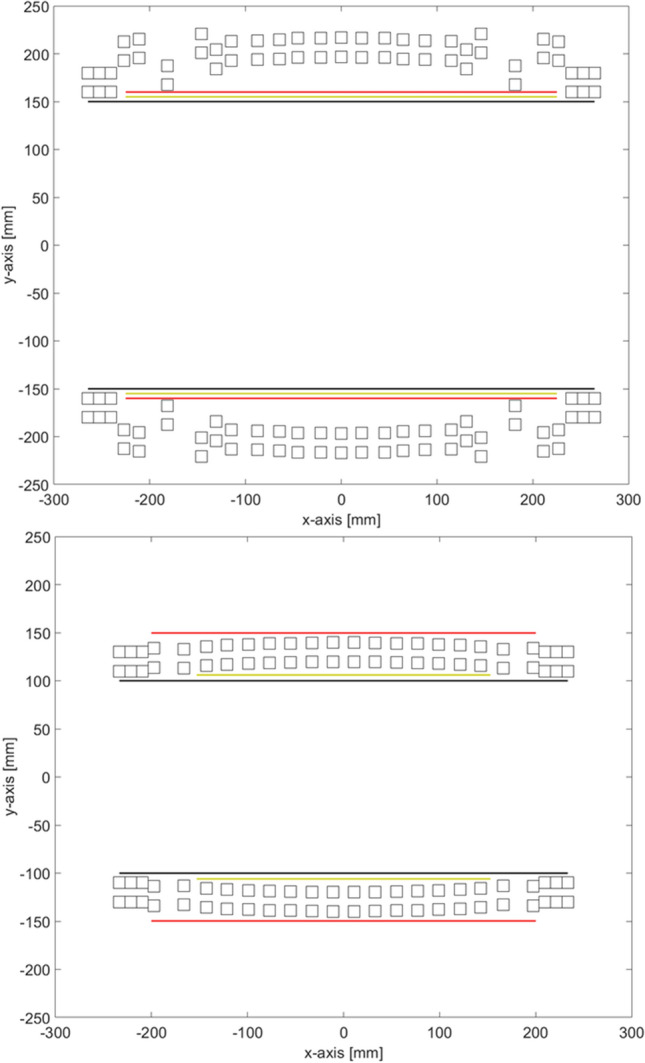
Side view of two magnet systems showing how the gradient coils fit into the design. The top figure corresponds to the final iteration of the neuroimaging system. The bottom corresponds to the extremity system. The blocks represent the magnet positions, red z-gradient, yellow *y*-gradient, black *x*-gradient. The top figure has a conventional layout, where the gradients are on the inside of the magnet. In the bottom figure the z-gradient is placed outside the magnet due to insufficient space on the inside

Figure 5 shows a side-view of the final configuration for both the neuroimaging and extremity system. The top figure corresponding to the neuro system shows that the gradient coils.fit inside the bore, while in the bottom figure the outer coil (*z*-gradient coil) needs to be placed at the exterior of the bore. This is because the homogeneity of the head system is restricted by the maximum length of the system, adding additional rings would create a more homogenous system, but will make it impossible for the head to reach the centre of the magnet. The extremity system requires one of the gradient coils to be placed exterior to the magnet in order to design a small enough magnet to reach 80 mT. An alternative approach would be to add an additional layer of magnets. However, this would increase the weight of the magnet significantly.

**Fig. 6:**
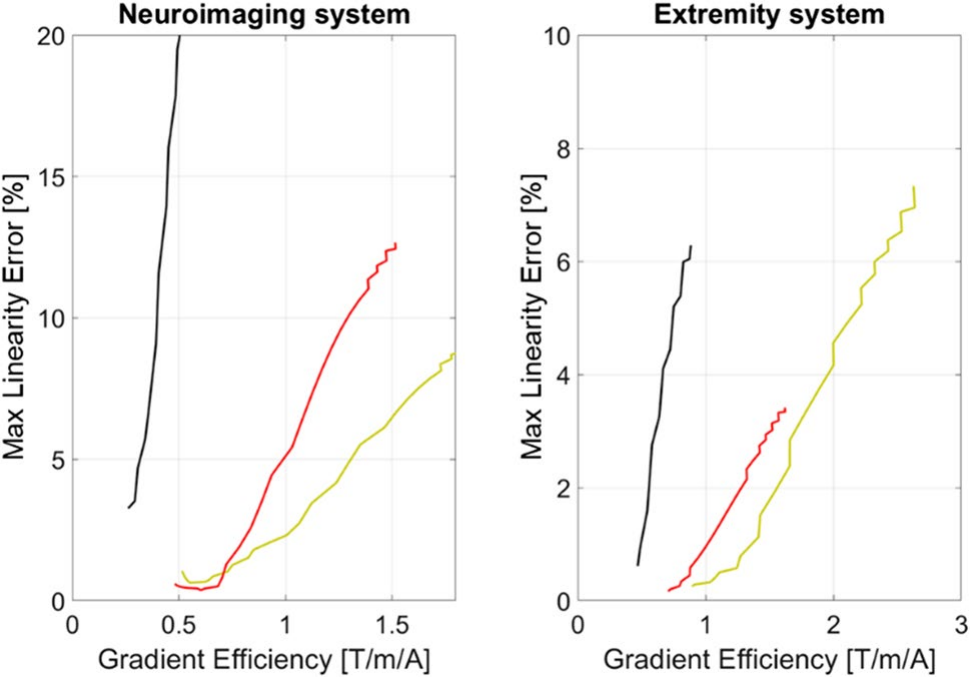
Linearity vs gradient efficiency plots for the three gradient coils. Linearity computed with respect to linear field inside the volume of interest. Red *z*-gradient, yellow *y*-gradient and black *x*-gradient

Figure 6 shows the linearity vs gradient efficiency plots for the final designs of the two systems. The curve of the *x*-gradient coil is the steepest meaning that an increase in efficiency is the most costly in terms of linearity. The gradient coils of the wrist system obtain the specified linearity more easily due to the dimensional difference between the systems.

## Discussion

The described framework gives the designer insight into how the magnet design influences the optimal gradient and RF geometries and consequently the imaging parameters that can be obtained with a specific setup. This is achieved with the integration of the time-efficient computational methods which determine the magnet, gradient and RF geometries. Integration has been shown to be important for dealing with the varying magnet ring-radii, which constrain the design space for the gradient coils and influences the obtainable spatial resolution. The two examples in the previous section show that systems for typical low-field point-of-care applications can be successfully designed, with the design of the neuro-imaging system similar in size and field strength to an earlier system [31]. The extremity system shows the importance of integrating the design methods, as the placement of one gradient outside the magnet allows sufficiently small ring-diameters to obtain the targeted field strength with a two-layer magnet design, while the combined performance of the gradient system and magnet homogeneity are sufficient to obtain the desired spatial resolution.

One of the strengths of the framework is that the target field methods described are compatible with any target region. The shape, location and values are the designer’s choice, the magnet, gradient and RF coils are all optimised for the specified region. This includes asymmetric designs which are not considered in this work but are within the framework’s capabilities.

In terms of the magnet design, the combination of ring-radii and -separation optimisation using a Newton algorithm shows the potential to be effective in obtaining highly homogenous magnet designs within short computational times compared to other numerical methods such as genetic algorithms. Some of the inputs requested from the designer, such as the starting number of rings, require some prior knowledge. This choice affects the amount of framework iterations necessary, but the initial conditions do not influence the final result, unless the number of starting rings is chosen larger than the minimum length solution, in which case the framework would finish after a single iteration. Another design choice is the B_0_ field strength, a sense for this parameter with a specified configuration can be obtained by solving the forward problem (3) using the minimum allowable radius. This will result in the maximum obtainable field strength of this configuration and gives the designer insight into the number of magnet layers required and the individual magnet strength to use.

In this work two magnet designs with a homogenous B_0_ field are discussed, it is also possible to consider a linear B_0_ field, effectively replacing one of the gradient coils. For a Halbach system the *y*- or *z*-gradient can be replaced by changing the fixed orientation of the magnets [27, 28].

The side views of the magnet rings in Fig. 5 show that the outer rings have reached their minimum spacing. If this constraint is removed, the model would produce overlapping rings. This indicates that an increase in magnetisation towards the ends of the magnet can further improve the homogeneity. One can consider adding an additional layer of magnets to the outer rings when this occurs [49].

Manufacturing errors have known to influence the realised homogeneity of Halbach systems [23, 28, 31]. There are a number of shimming methods that can be used to obtain the desired homogeneity [50, 51]. Consequently, the framework gives the homogeneity that needs to be obtained after shimming. Previous work has shown us that the gradient coil construction errors have less of an influence and that the magnetic fields modelled correspond closely with the measured fields created by the constructed coils [38].

In terms of gradient coil design there are alternative approaches which can optimize the gradient coil inductance [36]: requirements regarding switching speeds can easily be added into the framework. However, the greater challenge for compact, point-of-care systems with relatively low gradient amplifier current is typically to obtain sufficient homogeneity and linearity. Therefore, the emphasis is on optimising the power efficiency of the coils for a given linearity.

The blurring in the readout direction is determined with the least efficient gradient coil, to simulate the worst-case scenario and give the user flexibility in the choice of the readout-direction. In most cases the *x*-gradient is least efficient and therefore the choice could be made to design a system where the readout direction is always taken transverse to the bore. Consequently, the homogeneity and/or gradient amplifier requirements can be relaxed significantly.

The framework can be extended to include the effects of concomitant fields. These undesired vector components are unavoidable when creating the desired gradient fields, and can create distortions when the gradient field strength becomes significant with respect to B_0_ field strength [52–55]. One way to consider these effects is to constrain the dimensionless figure of merit $\epsilon =\frac{{G}_{max}{r}_{max }}{{B}_{0}}$, which gives an indication of the amount of distortions to expect. A more advanced way would be to consider the blurring and warping effects separately and take these into account when calculating the effective resolution and warping due to gradient non-linearities. Most models only account for the concomitant components of linear fields. Therefore, linearity must be constrained to a lower percentage for the models to be accurate, which can change the geometry of the coils. These effects are pulse sequence dependent and no research has been yet been published on concomitant fields specific for Halbach systems with cylindrical gradient coils.

The gradient coil heating is not modelled, but is an important factor that needs to be considered by the designer when choosing the wire thickness since excess heating can influence the strength of the permanent magnets or even damage them. There are a number of example systems in literature that can be referenced which use wire wound gradients [9, 10, 18, 28, 32, 33, 38, 41, 45].

The main RF coil considered in this work is the solenoidal coil, these coils have proven themselves for transverse magnetic field systems due to their superior sensitivity with respect to the saddle coil, which is the other single channel coil to consider [35]. The saddle coil however, has a better uniformity for a fixed length, which can be beneficial for short RF-coils.

## Data Availability

All data and code that support the findings of this study are available from the author, upon request.
